# Stand-Off Biodetection with Free-Space Coupled Asymmetric Microsphere Cavities

**DOI:** 10.3390/s150408968

**Published:** 2015-04-16

**Authors:** Zachary Ballard, Martin D. Baaske, Frank Vollmer

**Affiliations:** Max Planck Institute for the Science of Light, Laboratory of Nanophotonics & Biosensing, Guenther-Scharowsky-Str. 1/Bldg. 24, Erlangen D-91058, Germany; E-Mail: martin.baaske@mpl.mpg.de

**Keywords:** Whispering Gallery Mode (WGM) biosensors, free-space coupling, asymmetric resonant cavities, biosensing, label-free detection, protein adsorption

## Abstract

Asymmetric microsphere resonant cavities (ARCs) allow for free-space coupling to high quality (Q) whispering gallery modes (WGMs) while exhibiting highly directional light emission, enabling WGM resonance measurements in the far-field. These remarkable characteristics make “stand-off” biodetection in which no coupling device is required in near-field contact with the resonator possible. Here we show asymmetric microsphere resonators fabricated from optical fibers which support dynamical tunneling to excite high-Q WGMs, and demonstrate free-space coupling to modes in an aqueous environment. We characterize the directional emission by fluorescence imaging, demonstrate coupled mode effects due to free space coupling by dynamical tunneling, and detect adsorption kinetics of a protein in aqueous solution. Based on our approach, new, more robust WGM biodetection schemes involving microfluidics and *in-vivo* measurements can be designed.

## 1. Introduction

Optical microresonators have been demonstrated as powerful biodetection tools, recently even reaching single nanoparticle and single virus detection as well as monitoring specific interaction kinetics of single DNA oligonucleotides [1,2,3,4,5,6]. Efficient excitation of whispering gallery modes (WGM) in these demonstrations has been achieved by means of evanescent coupling utilizing, for example, tapered optical fibers and prisms [3,7,8,9]. Through positioning the microsphere resonators within the evanescent field, coupling efficiencies exceeding 99% have been observed with fiber tapers, up to 78% with prisms, and up to 28% with angle polished fibers [10,11,12]. However, despite excellent coupling efficiencies, biodetection measurements are particularly limited by the stability and incorporation of bulky and mechanically unstable fiber couplers [5,13,14,15]. Furthermore, fouling of the evanescent fiber couplers by the binding of particles and molecules to the taper adds unspecific background signals and degrades transmission through the taper [16]. This limits sensitivity and prolonged use of fiber coupled WGM biosensors. Although prism coupling has mitigated many of these challenges [3], it is necessary to explore entirely new coupling methods that do not rely on evanescent couplers, to optimize the design and effectiveness of future biodetection platforms [17,18].

One such method, free space coupling, relies on mode matching of a focused Gaussian beam for efficient excitation of high-Q WGMs in asymmetric cavities through a process called “chaos assisted dynamical tunneling” [19,20,21,22]. The creation of favorable coupling points along the perimeters of the resonator requires a break in its symmetry. Due to time-reverse symmetry, these coupling points also act as directional emission points, therefore enabling measurements of high-Q optical modes in the far field [23,24]. Also, the directional emission of asymmetric microavity lasers originates from these points. [24,25,26]. Directional emission from ARCs makes “stand-off” biodetection a possibility, where the necessary optics can be placed far away from the microresonator and measurements can be made in the far field. This scheme allows for a greater simplified incorporation into micro-fluidics and may enable novel in-vivo measurements. While free space coupling can also be achieved through the incorporation of engraved gratings, or though the placement of nano-scatterers along the resonator boundary, here we focus on the free space coupling mechanisms inherent in ARCs due to their ease of fabrication and simplified integration into any possible analyte solution for the purposes of biosensing [27,28].

The physics of free space coupling and directional emission has been previously studied, and high-Q free-space coupled resonators in air have been successfully demonstrated in several reports [22,29,30,31]. However, there has been little investigation into demonstrating free-space coupling in environments other than air, where achieving free space coupling in water is a particularly important goal for realizing biodetection with ARCs. Additionally, while reported coupling efficiencies for free-space coupled resonators have remained <10%, modes excited via free space have nonetheless been adequate for lasing and measurements with decent SNR, implying adequate coupling strength for the purposes of biodetection [23,27,28].

Here we demonstrate free space excitation of WGMs in ARCs immersed in water, which we use for label-free detection of protein adsorption. A tunable external cavity laser was used to excite the microcavities at ~405 nm wavelength to minimize the absorption of the light by water, allowing for efficient WGM excitation as well as far field detection of the light emitted from the cavity. To characterize the variations in directional emission of the fabricated ARCs in water, we devise a fluorescent imaging method to directly and comprehensively visualize ARC emission patterns. Placing a photodetector in the far field emission pattern, we then demonstrate stand-off WGM biodetection by monitoring adsorption kinetics of bovine serum albumin (BSA) onto a microsphere ARC resonator.

## 2. Directional Emission

### 2.1. The Ray Model for ARCs

Directional emission from ARCs enables measurements of optical modes in the far field. Directional emission has been studied extensively with various resonators geometries, including ellipse, half-circle-half-quadrupole (HCHQ), and even an egg shape resonator [18,22,29,30]. The boundary shape and degree of deformation have significant impact on the coupling and emission points as well as on the coupling efficiency to optical modes.

The mechanism for directional emission from a microcavity can be understood through a 2D chaotic billiard model. An ensemble of rays seeded into a microcavity with a given boundary and followed for a large number of internal reflections can be plotted in phase space, where each point identifies a reflection at the cavity boundary. The resulting map is known as a Poincare Surface of Section (PSOS) [21,32,33]. These plots are the stroboscopic view of a chaotic billiard system, where sinχ, the sine of the incident angle of any given reflected ray is plotted versus Φ, the polar angle at which the ray is reflected at the cavity boundary. These ray tracing simulations, have been studied extensively in the literature, and the directional emission behavior suggested by the theory has been measured experimentally in quadrupole and half-quadrupole-half-circular (HQHC) shaped boundaries [21,23,32,34,35]. By iteratively following the points in PSOS plots, indicating the successive reflection of  rays, it can be shown that chaotic ray trajectories evolve in phase space along well defined paths called “unstable manifolds” [32,33,36,37]. Each point along the unstable manifold is defined by the coordinates (Φ, sinχ), and can represent the point of refractive escape if sinχ equals sinχ_c_, the critical angle for internal reflection in a specific ARC. In other words, the unstable manifolds map the polar coordinates of refractive escape for a complete range of sinχ_c_ values. Ray diffusion along the path of unstable manifolds with resulting tangential emission is a good approximation for ARCs with small deformation. With small variation in sinχ for successive reflections, rays escape the cavity at angles very close to the critical angle and thus, as a result of Snell’s law, escape nearly tangent to the boundary [32].

**Figure 1:**
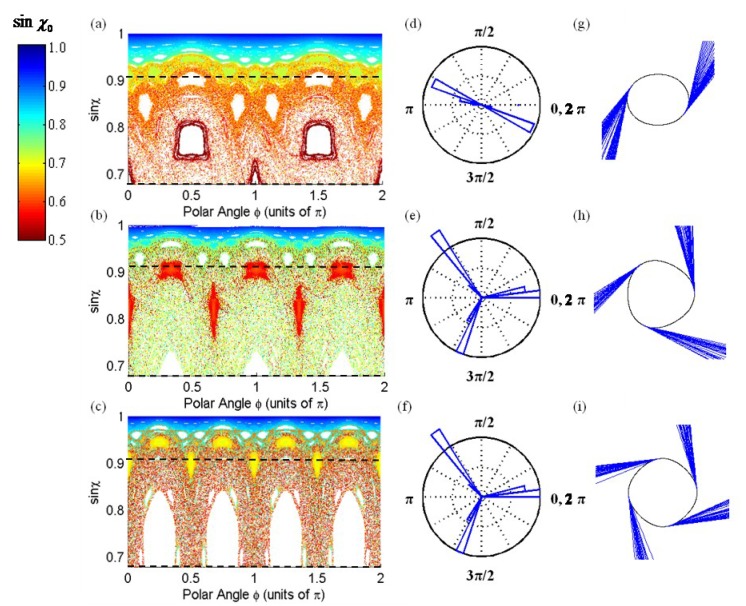
(**a**–**c**) PSOS plots for two, three, and four-pole boundary shapes where the color of each point corresponds to the initial sinχ_0_ value of the injected ray. For this simulation 250 rays were seeded into the 2-D boundaries at π/2 with an initial linear angular spread from 45° to 90° and followed for 3000 reflections. The dotted lines at sinχ = 0.68 and sinχ = 0.91 indicate the sinχ_c_ for a SMF-28 (n~1.47) resonator in air and water respectively; (**d**–**f**) histogram plots of the polar angle at which rays escape refractively from the cavity in air (**g**–**i**) the corresponding ‘m-pole’ boundary shape with the exiting rays from the chaotic billiard simulation.

We examine the angles of refractive escape from 2D disk-shaped microcavities where the boundaries are defined by the quadrupole polar equation:

(1) $$R\left(\phi \right)=\frac{R_{0}\left(1+\varepsilon \cos\left(m\phi \right)\right)}{\sqrt{1+\varepsilon^{2}/2}}$$

where ε is the degree of deformity ε = (R_a_ – R_b_)/(R_a_ + R_b_), R_a_ and R_b_ are the length of major and minor axis respectively. R_0_ is the non-deformed radius, and *m* is the number of poles in the boundary [31,38]. For the ray-tracing simulation in Figure 1, sinχ_c_ was set as 0.68, to simulate a fused silica resonator, *i.e.*, SMF-28 glass of index n~1.47 in air. R_0_ is arbitrary to the directional emission patterns and thus defined as 1, and ε was defined as 0.1, 0.05, and 0.025 for the 2-pole, 3-pole, and 4-pole ARCs respectively. As shown in Figure 1, using this equation, the number of poles directly impacts the number of distinct emission points, and for these conditions, the unstable manifolds intersect sinχ_c_ at polar angles (Φ_emission_) corresponding to the points of highest curvature along the resonator boundary. Thus rays exclusively escape from these points of highest curvature, *i.e.*, where the poles exist.

However, if the resonator is placed in a higher index environment such as water (n ~ 1.34), the sinχ_c_ value increases, which causes changes to the directional emission strength. This is due to the fact that fewer of the rays seeded at random sinχ values (and fixed coupling angle ϕ = π/2) at the onset of our PSOS calculation are trapped by total internal reflection and guided via chaotic trajectories to particular emission points. In other words, an ARC with a low sinχ_c_ value due to immersion in a lower index environment enables a greater number of randomly seeded rays to propagate via total internal reflection in the ray-tracing simulations. As illustrated in Figure 1, the critical angle cut-off for SMF-28 in air (sinχ_c_~0.68) enables a much larger region of the chaotic sea to exist within the resonator as opposed to the critical angle cut-off for SMF-28 in water (sinχ_c_~0.91). For SMF-28 in air, a larger fraction of randomly seeded rays thus participate in the “chaotic sea” and are guided via the unstable manifolds to their directional emission points. As observed from our simulations, this results in stronger directional emission patterns when comparing the ARC in low index environments versus the ARC in higher index environments, and suggests more efficient free space coupling when comparing the former to the latter.

**Figure 2:**
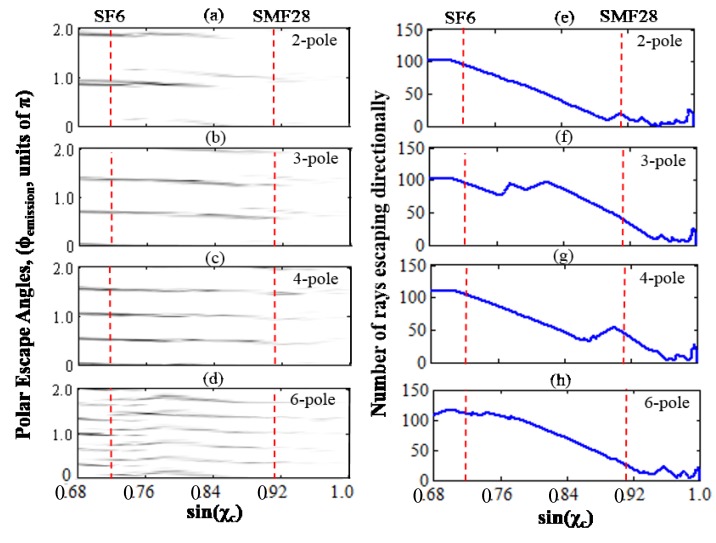
Further analysis of the ray-tracing simulation. (**a**–**d**) shows the polar escape angles (ϕ_emission_) of the rays as a function of the critical angle parameter (sinχ_c_). The gray value indicates the number density of the rays guided via chaotic trajectories to directional emission points; (**e**–**h**) shows the total number of rays escaping at directional emission points for a certain sin(χ_c_) in the ray-tracing simulation of 250 randomly seeded rays (*i.e.*, the integration of (a–d) along ϕ) The dotted red lines indicate the critical angles for SF6 glass in water (sinχ_c_~0.72) and SMF-28 glass (sinχ_c_~0.91) in water.

By further analyzing the results of the ray-tracing simulation, the number of rays which are trapped by total internal reflection and guided to directional emission points can be determined as a function of sinχ_c_, Figure 2.

Therefore, the ray model seems to suggest that in order to maintain strong directional emission and thus, in reverse, efficient free-space coupling in higher index environments, the ARC resonator refractive index should be raised in order to decrease the sinχ_c_ value. In our simulations, this allows for more rays to be trapped in the chaotic sea by total internal reflection and guided to emission points. Specifically, for biodetection measurements, the SMF-28 silica resonators (n ~ 1.47) in water seems unfavorable, because the high sinχ_c_ value supports only a small fraction of seeded rays to be guided via total internal reflection to the directional emission points. Thus a higher index glass such as SF6 (n ~ 1.86) should be more suitable for practically achieving more efficient free-space coupling in aqueous environment. Furthermore, SF6 can provide cavities with a material limited Q factor of ~1.3 × 10^7^, and a laser source exhibiting minimal absorption losses in aqueous environment when operating at ~405 nm nominal wavelength, close to the absorption minimum of water, can be used to maintain high Q factors even after immersion in water.

### 2.2. Visualizing Directional Emission Patterns from ARCs

To test the trends predicted by our simulations, microspheres were fabricated by melting standard SMF-28 fibers (n ~ 1.47) as well as SF6 fibers (n ~ 1.86) into spherical shapes with diameters of 30–80 μm. Then, slight deformations were introduced to the spheres by a series of 10–20 ms pulses from a focused 30 W CO_2_ laser operating at 20% of its duty cycle [23]. Typically, to induce an effective deformation, resonators were pulsed two to three times on two opposite sides. Deformation parameters were then resolved with a 10× imaging system, and analyzed with Image *J*. For this specific study, ARCs were fabricated with a deformation parameter of ε < 2%. However, because the deformation is small (ε < 2%), accurately defining a function describing the boundary shape of the ARC is very difficult with optical imaging alone. We therefore implement an approach based on fluorescence imaging to directly visualize the emitted light pattern of the ARC, identify the number of poles, and thus assign a theoretical boundary shape that can predict the observed emission properties.

**Figure 3:**
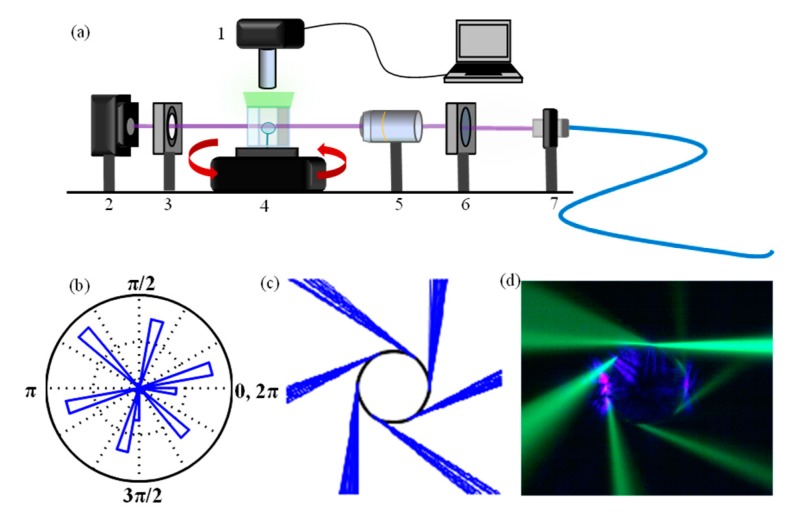
(**a**) Experimental set-up for visualizing directional emission of ARCs in aqueous solution: 1. Imaging system 2. Photodetector for measuring modes in the far field 3. Spatial filter for isolating far field emission pattern. 4. Rotation stage, with ARC and chamber with GFP in PBS buffer 5. The 10× objective is used for focusing the coupling beam 6. Quarter-wave plate for polarization control 7. Fiber collimator (**b**) Polar histogram of refractive escape for 6-pole boundary ARC from (**c**) simulation of refractive escape of rays injected into the ray-tracing simulation (ε = 0.01) (**d**) real color image of directional emission by GFP fluorescence imaging of 63 μm diameter ARC fabricated from SF6 fiber.

For establishing fluorescence imaging of the emission pattern, ARCs were submerged in chambers of 10 µg/mL Green Fluorescent Protein (GFP) (Active *A. victoria* GFP full length Protein ab84191) in a PBS buffer (pH 7.0). The beam waist of the 405 nm laser was brought just to the outside of the resonator boundary, and the resonator was rotated to the optimal coupling spot along the circumference. An imaging system directly above the resonator was then able to capture the ~509 nm fluorescent emission from the GFP, thereby spatially resolving the directional emission pattern as well as the incoming beam (Figure 3).

We tested our hypothesis of maintaining directional emission in higher index environments through the use of higher index resonators made from SF6 fiber as compared to those made from SMF-28 fiber. Once free space mode coupling to our SF6 resonators was confirmed in air, see also next Section 3, the GFP solution was injected into the chamber and the fluorescence signal was imaged. Figure 3d is, to the best of our knowledge, the first direct, comprehensive experimental visualization of directional emission due to the chaotic nature of ray-dynamics. The observed emission pattern can be recreated with the ray-tracing simulation by implementing a boundary with six poles and deformation parameter (ε) of 0.01. For all the SF6 resonators fabricated, emission patterns with six or seven poles were observed by imaging. Consistent with the trends for coupling efficiency predicted by our PSOS plots, we only observe excitation of higher Q resonances (WGMs) with SF-6 ARCs in water, as we will show in the following, and report no observation of microcavity resonances of significant Q factors for free space coupling to SMF-28 ARCs after their immersion in an aqueous environment. This positive result for SF6 agrees with the hypothesis made in Section 2.1, but the high number of poles in the boundary is surprising. Most literature up to date, which has not yet directly imaged the emission pattern, has only measured ARCs with either two or four emission directions in the far field theoretically defined by quadrupole or half-quadrupole-half-circle boundaries [21,35,39,40]. However, the observed 6‒7 pole emission was repeatable and can be explained due to a resonator boundary with a six pole shape, created through the CO_2_ laser pulse technique, where each laser pulse creates a unique dent in the boundary responsible for a certain number of distinct poles [23]. Next, we placed our photodetector in the imaged far field emission patterns (in pure water) to measure modes in the far field.

## 3. Measuring Modes in the Far-Field

The ray-optics model can be used to explain the directional emission of an ARC, however, it forbids propagating rays to jump between regions bound by the Kolmogorov-Arnold-Moser (KAM) invariant curves [21,25,32,33]. These KAM curves separate regions of phase space, classically forbidding chaotic trajectories of rays to diffuse into other, higher regions of phase space where more WGM-like ray trajectories occur. These separated regions are illustrated by the regions of distinct color in the PSOS plots in Figure 1. Therefore the lower Q-factors of modes which may be emitted refractively from the chaotic (lower) region of KAM curves are fundamentally limited by the mean classical lifetime of the photons. However, despite these KAM curves, high-Q modes, such as WGMs, can still be measured in the far field of ARCs. Free-space excitation of high Q WGMs in ARCs is possible due to “chaos assisted dynamical tunneling,” and is evidenced form the observation of coupled mode effects [19,20,21,22,31,39], which we explain in the following and which are supported by our experimental data.

In free-space coupling, no coupler exists in the evanescent field, therefore high-Q optical modes are not able to tunnel out of the resonator via a fiber or prism. They therefore rely on chaos-assisted dynamical tunneling as their primary escape mechanism. This phenomenon thus can be explained as the interference between two separate coupling pathways. The first pathway occurs when modes are excited directly in the chaotic region of phase space through light which has refracted into the resonator from the focused beam. The second pathway occurs when high-Q modes, excited via chaos-assisted tunneling, couple back into the chaotic region of phase space. When the chaos-assisted dynamical tunneling occurs between the regions separated by the KAM curves, a phase shift occurs, and this results in far field emission spectra with interfering high Q and low Q modes [21,41]. This interference has been shown to exhibit different lineshapes such as Fano-resonances- and Electromagnetically Induced Transparency (EIT)-like line shapes known as chaos-induced transparency [20,21,22,31,32,41].

**Figure 4:**
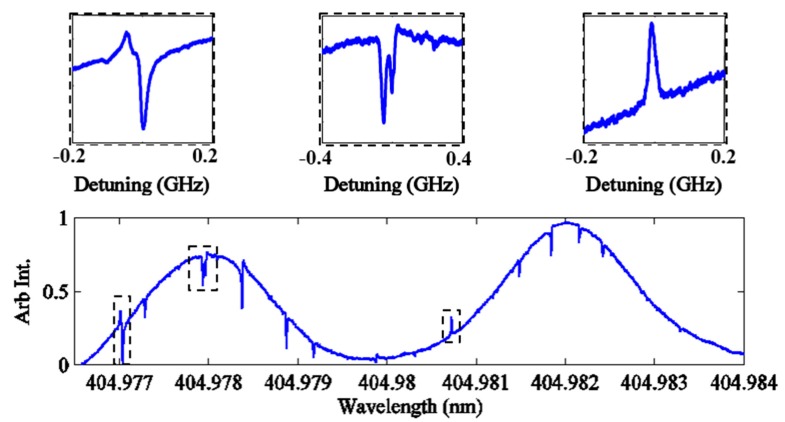
Swept wavelength spectrum obtained for a 60 µm diameter SMF-28 ARC in air. (**a**–**c**) shows lineshapes of high-Q-modes embedded in the low-Q modes appearing as peaks in the full transmission spectrum of the far-field ARC emission depicted in (**d**).

Resonances with Q > 10^7^ embedded in lower Q~10^5^ resonances were routinely measured for microsphere resonators in air fabricated for this study, Figure 4. Furthermore, the observed Fano- and EIT- like lineshapes exhibit the coupled mode behavior that is expected for chaos assisted tunneling between high and low Q modes.

Excitation of modes exhibiting Q factors of only ~10^4^‒10^5^ were observed after immersing the SF6 ARC resonators in aqueous environments. On the other hand, no such modes were observed for SMF-28 ARCs after immersion in water, following the trends predicted by our ray-tracing simulations. The absence of much higher Q modes for SF6 in water and no appearance of spectral features after water immersion that could indicate mode coupling effects may be due to weaker directional emission from the slightly higher critical angle of SF6 in water (sinχ_c_ = 0.72) as compared to SMF-28 in air (sinχ_c_ = 0.68). Water absorption could also play a role in lowering the coupling rate in and out of the resonator. Nevertheless, the lower Q modes observed for SF6 in water had a strong enough signal to noise ratio to demonstrate a simple biodetection measurement which we report next.

## 4. Biosensing with Free-Space Coupled ARC

SF6 ARC resonators were fabricated, aminosilianized [42], then submerged in PBS buffer. While the ARCs were in the buffer, the emission was spatially filtered and modes were detected by a photodetector placed in the far field. Then bovine serum albumin (BSA) protein was injected into the chamber, away from the resonator, resulting in an equilibrium concentration of 0.5 μM. Spectra were obtained at a rate of 11.9 Hz by swept wavelength scanning during the protein adsorption process, and a parabolic fit was used to track the optical mode (Q ~ 10^5^) and extract the shift of the resonance wavelength [43].

**Figure 5:**
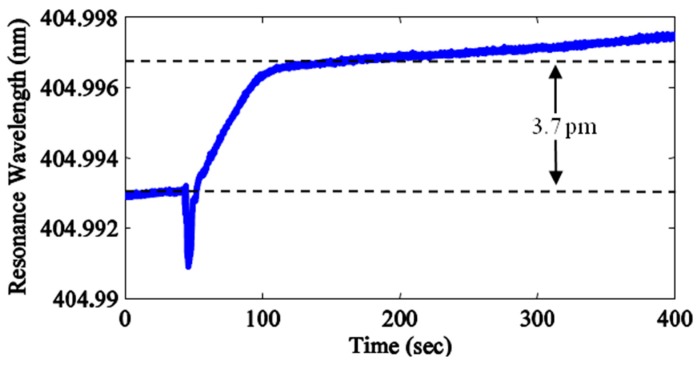
BSA adsorption curve measured in the far-field of a free-space coupled ARC fabricated from SF6 glass with a diameter of 68 μm.

Figure 5 demonstrates biodetection with a free-space coupled microsphere ARC resonator. The resonance shift δλ and excess polarizability α_ex_ of the BSA protein can be used to estimate σ_s_, the surface density of adsorbed BSA protein [38]. The transient negative wavelength shift indicates injection of the protein solution. At equilibrium, an overall positive wavelength shift of δλ ~ 3.7 pm was observed after ~400 s of incubation time, which follows the equation:

(2) $$\frac{\delta \lambda }{\lambda }\approx \frac{\alpha_{ex}\sigma_{s}}{\varepsilon_{0}({n_{1}}^{2}-{n_{2}}^{2})R}$$

where α_ex_(BSA) ~ 4πε_0_ × 4.322 × 10^−21^ cm^3^, ε_0_ is the permittivity of free-space, n_1_ and n_2_ are the refractive indices of the sphere and the buffer solution, respectively, R is the nominal ARC microsphere radius (34 μm), and λ the nominal wavelength of the laser (~405 nm). From Equation (2), we estimate the surface density for the ~3.7 pm resonance wavelength shift measured after protein adsorption at σ_s_ ~ 9.52 × 10^11^ cm^−2^. This corresponds to about 35% of the surface density expected for a monolayer of BSA [40] and thus our results indicate the formation of an incomplete but single layer.

## 5. Conclusions

Free-space coupled ARC WGM biodetectors can provide an advantage over evanescently coupled prism or fiber coupled resonators due to their un-tethered setup. Therefore work into understanding the limitations and mechanics of this new coupling scheme is needed for realizing innovation and more sensitive biodetection platforms.

This study has detailed a novel visualization procedure for studying directional emission of ARCs immersed in aqueous environments, and has also demonstrated the first example of a free-space coupled biodetection measurement, proving the possibility for realizing a sensitive, practical ARC stand-off biodetection platform. By better understanding the mechanics of free-space coupling and directional emission of ARCs in higher index environments, stand-off biosensing platforms can be optimized and utilized in unique detection schemes, perhaps in-vivo, where evanescent coupling set-ups with prisms or fibers are not feasible. Furthermore the fano and EIT-like resonances in free-space coupled resonator spectrum could be used to enable increased sensitivity and improved signal to noise in biodetection measurements by utilizing multi-spectral analysis [44,45].

Furthermore, through the engineering and simulation of boundary shapes, specific directional emission patterns can be realized as a customization for stand-off detection, allowing flexibility based on the proposed application. During the ARC fabrication procedure, the number and placement of laser pulses could be used to induce local deformation and thus create ARCs with specific m-pole emission patterns. For example, if ARCs were engineered to emit strongly in the backwards direction, then the focusing objective could also be used to efficiently capture and measure the optical modes, thus acting as a simple bio-sensing platform design. Also, for multiplexed detection, resonators with several emission points could be incorporated so that optical modes were sent to an array of detectors in the far field.
